# A new basic science journal for ESSKA

**DOI:** 10.1186/s40634-014-0006-9

**Published:** 2014-06-26

**Authors:** Jon Karlsson, João Espregueira-Mendes, C Niek van Dijk

**Affiliations:** 1 Department of Orthopaedics, Sahlgrenska University Hospital, Gothenburg, Sweden; 2 Orthopaedic Department of Minho University, Braga, Portugal; 3 European Society of Sports Traumatology, Knee Surgery and Arthroscopy, Spain; 4 Education Committee of the International Society of Arthroscopy, Knee Surgery and Orthopaedic Sports Medicine, France; 5 Mendes Sports Medicine Centre, FIFA Medical Centre of Excellence, Spain; 6 Porto University, Porto, Portugal; 7 Minho University, Braga, Portugal; 8 Department of Orthopaedic Surgery, Academic Medical Center, University of Amsterdam, Meibergdreef 9, Amsterdam, 1105 AZ The Netherlands

**Keywords:** ■■■

ESSKA is a non-profit organization that stands for quality, independence, high ethical standards and rigor.

ESSKA has during the past few years experienced an exceptional growth, undergoing several important changes/improvements and consolidating its position as one of the world’s leading societies in orthopaedic sports traumatology and degenerative joint disease.

ESSKA has doubled the number of members in the past two years and with its 2800 members and 26 affiliated societies ESSKA is the most important European sports traumatology association.

ESSKA has during the last 10 years taken increased responsibility for research and education. The ESSKA journal; Knee Surgery Sports Traumatology Arthroscopy (KSSTA) is now more than 20 years and is increasingly popular as the clinical journal of ESSKA. But, we must remember that all clinical and patient-related research is based on basic science.

Why a new Journal? Basic science is the corner-stone for the understanding of disease on which depends the delivery of high quality patient care. This is what Evidence-Based Medicine (EBM) is founded upon. Whilst Europe has a very strong position in terms of research and science, it was perceived to be lacking an outlet for its the basic reserach scientists (within the field of Orthopaedic Sports Medicine/Traumatology and degenerative joint diseases) to disseminate their findings. This gap, (in ESSKA’s view), therefore warranted the setting up of a basic science journal.

A development strategy set out during a strategic planning meeting in Amsterdam 2010 by ESSKA’s Board, highlighted and addresssed (over the intervening years), the development needs of such a scientific journal, alongside with KSSTA. Following the initial preparatory work, the journal was successfully launched in December 2013 under the leadership of Henning Madry. Springer will publish the new journal with the Journals’editorial office based in Luxembourg.

The new journal, entitled the Journal of Experimental Orthopaedics (JEO) shall collaborate closely with the Knee Surgery Sports Traumatology Arthroscopy journal (KSSTA), ESSKA’s clinical journal. The intention being, to build a strong publication team in order to cover the entire scientific field of sports traumatology and degenarative joint desease.

This is a similar approach to that which AOSSM developed with their successful American Journal of Sports Medicine (AJSM) and now Orthopaedic Journal of Sports Medicine (OJSM). Also AANA recently added Arthroscopic Techniques to their already successful journal of Arthroscopy.

As mentioned before ESSKA found the need to move in this same direction and responded with the development of the JEO. The ESSKA Board saw that the most important contribution would be to basic science within the field of orthopaedic sports traumatology and degenerative joint desease. ESSKA’s reputation stands for quality patient care through education and this new journal will reflect and build on the proud reputation of KSSTA these core values.

In line with this new relationship between the KSSTA and JEO journals, the ESSKA Board has decided that KSSTA will in the future concentrate mainly on clinical topics. The Editors in Chief of both journals are fully aware that this transition will take some time and therefore it is expected that the number of experimental/basic science studies in KSSTA will reduce in number over a period of time and in the future any such studies submitted to KSSTA shall be cascaded to JEO; this being optional to authors, of course. In this manner the sister journals will be better able to cover the full spectrum of basic science and clinical studies in our field in the future.

JEO will be published as an open access, online publication benefiting from fast handling of manuscripts and on completion immediate publication. JEO’s prevailing aim is to bridge the gap between basic science and the clinical field and welcomes basic science studies on all aspects of musculo-skeletal diseases.

We welcome the new journal and we invite you to submit your manuscripts to the JEO at; www.jeo-esska.com.

